# Predictive Modeling for Voxel-Based Quantification of Imaging-Based Subtypes of Pancreatic Ductal Adenocarcinoma (PDAC): A Multi-Institutional Study

**DOI:** 10.3390/cancers12123656

**Published:** 2020-12-05

**Authors:** Mohamed Zaid, Lauren Widmann, Annie Dai, Kevin Sun, Jie Zhang, Jun Zhao, Mark W. Hurd, Gauri R. Varadhachary, Robert A. Wolff, Anirban Maitra, Matthew H. G. Katz, Joseph M. Herman, Huamin Wang, Michael V. Knopp, Terence M. Williams, Priya Bhosale, Eric P. Tamm, Eugene J. Koay

**Affiliations:** 1 Department of Radiation Oncology, The University of Texas MD Anderson Cancer Center, Houston, TX 77030, USA; mmzaid@mdanderson.org (M.Z.); LWidmann@mdanderson.org (L.W.); Annie.Dai@bcm.edu (A.D.); KSun1@mdanderson.org (K.S.); joeradonc@gmail.com (J.M.H.); 2 Department of Experimental Radiation Oncology, The University of Texas MD Anderson Cancer Center, Houston, TX 77030, USA; JZhang27@mdanderson.org; 3 Sheikh Ahmed Center for Pancreatic Cancer Research, The University of Texas MD Anderson Cancer Center, Houston, TX 77030, USA; JZhao6@mdanderson.org (J.Z.); MWHurd@mdanderson.org (M.W.H.); 4 Department of Gastrointestinal Medical Oncology, The University of Texas MD Anderson Cancer Center, Houston, TX 77030, USA; gvaradha@mdanderson.org (G.R.V.); rwolff@mdanderson.org (R.A.W.); 5 Department of Pathology, The University of Texas MD Anderson Cancer Center, Houston, TX 77030, USA; amaitra@mdanderson.org (A.M.); hmwang@mdanderson.org (H.W.); 6 Department of Surgical Oncology, The University of Texas MD Anderson Cancer Center, Houston, TX 77030, USA; mhgkatz@mdanderson.org; 7 Department of Radiology, The Ohio State University Wexner Medical Center, Columbus, OH 43210, USA; Michael.Knopp@osumc.edu; 8 Department of Radiation Oncology, The Ohio State University Wexner Medical Center, Columbus, OH 43210, USA; terence.williams@osumc.edu; 9 Department of Abdominal Imaging, The University of Texas MD Anderson Cancer Center, Houston, TX 77030, USA; priya.bhosale@mdanderson.org (P.B.); etamm@mdanderson.org (E.P.T.)

**Keywords:** pancreatic cancer, computed tomography, imaging biomarker, radiomics, machine learning

## Abstract

**Simple Summary:**

Previously, we demonstrated that qualitative scoring of pancreatic ductal adenocarcinoma (PDAC) tumors on computed tomography (CT) scans (delta) is biologically and clinically relevant, whereby tumors with a conspicuous border (high delta) show more aggressive biology and are associated with worse clinical outcomes when compared to those with an inconspicuous border (low delta). However, in some cases, a visual classification can be challenging and subjective. Here, we used machine learning and quantitative approaches for a multi-institutional dataset to build a biologically and clinically relevant model that can quantitatively identify these imaging-based subtypes of PDAC from routine CT scans. Our results showed that the quantitative classification (q-delta) had high correlation with the gold standard qualitative scoring in internal and external datasets. Further, q-delta classification was demonstrated to be associated with the clinical outcome and the stromal heterogeneity of the PDAC tumors. High intra- and interrater agreement scores indicate the reproducibility of the results.

**Abstract:**

Previously, we characterized qualitative imaging-based subtypes of pancreatic ductal adenocarcinoma (PDAC) on computed tomography (CT) scans. Conspicuous (high delta) PDAC tumors are more likely to have aggressive biology and poorer clinical outcomes compared to inconspicuous (low delta) tumors. Here, we developed a quantitative classification of this imaging-based subtype (quantitative delta; q-delta). Retrospectively, baseline pancreatic protocol CT scans of three cohorts (cohort#1 = 101, cohort#2 = 90 and cohort#3 = 16 [external validation]) of patients with PDAC were qualitatively classified into high and low delta. We used a voxel-based method to volumetrically quantify tumor enhancement while referencing normal-pancreatic-parenchyma and used machine learning-based analysis to build a predictive model. In addition, we quantified the stromal content using hematoxylin- and eosin-stained treatment-naïve PDAC sections. Analyses revealed that PDAC quantitative enhancement values are predictive of the qualitative delta scoring and were used to build a classification model (q-delta). Compared to high q-delta, low q-delta tumors were associated with improved outcomes, and the q-delta class was an independent prognostic factor for survival. In addition, low q-delta tumors had higher stromal content and lower cellularity compared to high q-delta tumors. Our results suggest that q-delta classification provides a clinically and biologically relevant tool that may be integrated into ongoing and future clinical trials.

## 1. Introduction

Despite the tremendous efforts and resources invested in enhancing the diagnostics and therapeutics of pancreatic ductal adenocarcinoma (PDAC), it continues to demonstrate rising incidence and to pose high mortality [1]. The lethality of PDAC can be attributed to late diagnosis, tumor resistance to chemotherapy and radiation therapy, and the complexity of the tumor microenvironment [2,3]. Hence, there is a growing need for a robust biomarker that could objectively address the heterogeneity of the PDAC tumors and identify those who can benefit from certain therapeutic strategies more than others. While CA 19–9 remains the only Food and Drug Administration-approved prognostic biomarker for PDAC, it is still limited to patients with the Sialyl Lewis a–positive genotype and normal serum bilirubin levels [4]. 

Previously, we developed a normalized area under the curve (nAUC) method to characterize the mass transport properties of PDAC tumors through systematic sampling of the tumors and normal pancreas using pancreatic protocol CT scans. nAUC significantly correlated with gemcitabine delivery, radiation response, stromal reaction, and clinical outcomes [5,6]. In addition, we showed that qualitative visual scoring of the change in enhancement on CT scans at the interface between PDAC tumors and parenchyma (delta) is biologically and clinically relevant, whereby tumors with a conspicuous border (high delta) possess lower degrees of stroma, show more aggressive mesenchymal biology, are more likely to have multiple common pathway mutations, and are associated with worse clinical outcomes compared to those with an inconspicuous border (low delta) [7,8,9,10,11]. However, in borderline cases, a qualitative classification can be challenging to the reader, and the presence of a quantitative metric that can objectively identify the delta class may help with clinical integration.

We hypothesized that a commercially available voxel-based quantitative-enhancement feature, known as qEASL (quantitative European Association for the Study of the Liver), would provide this quantitative metric. The qEASL metric has been utilized as an imaging biomarker of response in patients with liver cancer [12,13,14]. Within a 3D-segmented structure, qEASL quantifies the number of voxels that enhance (*n*) standard deviations of the referenced normal voxels [15].

In this multi-institutional study, we used machine learning and quantitative enhancement features of PDAC tumors, derived from pre-therapy pancreatic protocol CT scans, to build a clinically relevant model that can quantitatively classify these tumors. We called this classifier “quantitative delta” (q-delta). Furthermore, we hypothesized that this classification would show clinical and histopathological differences.

## 2. Results

### 2.1. Patient Demographics

Patients in the training set (D1) consisted of 55 males (54%) and 46 females (46%) the median age was 64 years (range = 25–85 years), and the median overall survival was 25 months. In total, 17 patients had stage I, 32 had stage II, and 52 had stage III tumors. The internal validation set (D2) consisted of 52 males (58%) and 38 females (42%); the median age was 63.25 years (range = 42–82 years), and the median overall survival was 21.3 months. In total, 65 patients of the D2 cohort underwent surgical resection; 31 patients had stage I, and 25 had stage II, and nine had stage III tumors. The external validation set (D3) consisted of 10 males (63%) and six females (37%); the median age was 66 years (range = 34–88 years). In total, 11 patient had stage II and five patients had stage III tumors (Table 1). Staging was based on the 8th edition of the American Joint Committee on Cancer (AJCC) guidelines for PDAC.

It is noteworthy to mention that there were no significant associations between the gender of the patient and the q-delta class in D1 (Likelihood ratio, *p* = 0.07), D2 (Likelihood ratio, *p* = 0.09), and D3 (Likelihood ratio, *p* = 0.2).

### 2.2. Association between Qualitative Delta and q-delta

**Figure 1:**
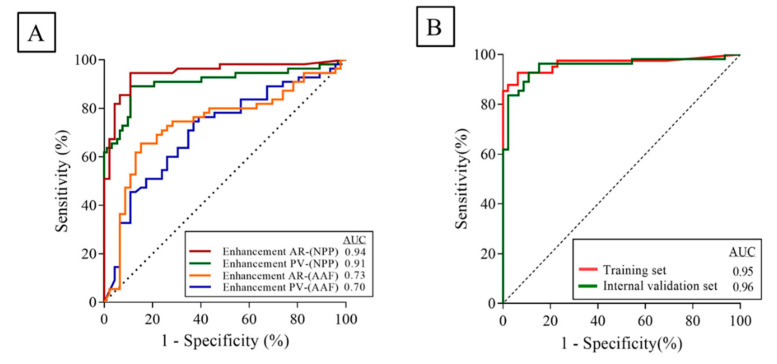
(**A**) Univariate logistic regression analysis of the measured quantitative enhancement values versus the qualitative delta scoring using the training data set. (**B**) Multivariate logistic regression analysis using the model identified measurements (q-delta), in training D1 (*n* = 101), and internal validation D2 (*n* = 90). Abbreviations: (AR)-NPP: enhancement at the arterial phase normalized to normal pancreas parenchyma; (PV)-NPP: enhancement at the portovenous phase normalized to normal pancreas parenchyma; (AR)-AFF: enhancement at the arterial phase normalized to anterior abdominal fat; (PV)-AFF: enhancement at the portovenous phase normalized to anterior abdominal fat.

To identify the association between the obtained NE values and the qualitative delta scoring, we performed univariate, followed by multivariate nominal logistic regression analyses using D1 (*n* = 101). The univariate analysis showed significant associations between the qualitative delta scoring and the NE values obtained at (1) the AR phase while referencing NPP (AUC = 0.94, 95% CI = 0.90–0.99, *p* < 0.0001), (2) AR phase while referencing AAF, (AUC = 0.73, 95% CI = 0.63–0.84, *p* < 0.0001), (3) PV phase while referencing NPP (AUC = 0.91, 95% CI = 0.84–0.97, *p* < 0.0001), and (4) PV phase while referencing AAF (AUC = 0.70, 95% CI = 0.60–0.81, *p* = 0.0003)) (Figure 1A).

A backward elimination process identified (1) NE at the AR phase while referencing NPP (*p* = 0.03) and (2) NE at the PV phase while referencing NPP (*p* = 0.007) as the most significant parameters and were subsequently used to build a predictive model (q-delta model) using multivariate logistic regression analysis. The model used a probability formula to output the most likely response; high quantitative delta (q-delta) versus low q-delta (Figure S1), and it achieved a significantly high area under the curve (AUC) of 0.95 (95% CI = 0.91–0.99, *p* < 0.0001)**.**

To test the accuracy of the model, we computed the ROC for D2 (*n* = 90) and D3 (*n* = 16) using the parameters and the probability formula identified in the training set. D2 achieved a significantly high AUC of 0.96 (95% CI = 0.92–1, *p* < 0.0001), and D3 achieved significantly high sensitivity (86%) and specificity (100%) in identifying the binary delta class (Figure 1B). Results of the univariate and multivariate analyses are shown in Table 2.

### 2.3. Association between Quantitative Delta and nAUC

In the three studied cohorts, there were significant correlations between the nAUC and the normalized enhancement values in D1 (ρ = 0.5, *p* < 0.0001), D2 (ρ = 0.4, *p* < 0.0001), and D3 (ρ = 0.6, *p* = 0.006). In addition, there was a significant association between nAUC and the q-delta classes in D1, D2, and D3 (*p* < 0.0001, *p* < 0.0001, and *p* = 0.007, respectively; (Figure S2).

### 2.4. Inter- and Intrarater Variability Assessment

DSC showed good inter- (mean = 0.86 ± 0.03) and intrarater (mean = 0.9 ± 0.02) agreement rates for the PDAC tumor contours. The ICC model showed good and excellent interrater agreement rates for the quantified NE values at the AR (0.84, 95% CI: 0.71–0.91) and PV (0.90, 95% CI: 0.82–0.95) phases, respectively. Similarly, the ICC model showed excellent intrarater agreement rates for the quantified NE values at the AR (0.95, 95% CI: 0.92–0.97) and PV (0.93, 95% CI: 0.87–0.96) phases (Table S1). The inter- and intrarater repeat measurements achieved 90% overall accuracy in predicting the qualitative delta class.

### 2.5. Association between the q-delta and Tumor Stromal Content

**Figure 2:**
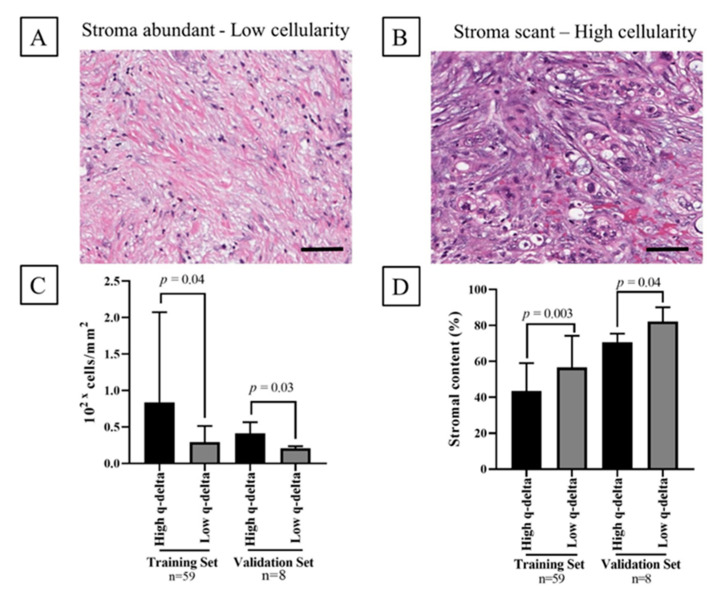
(**A**,**B**) representative H&E images of the different stromal content. Scale bar = 50 μm (**C**,**D**). show the association between q-delta classification and the stromal cellularity in 59 patients from the training set (D1) and eight patients from the validation (D3) who had pathology specimens available for analysis.

T-test revealed a significant association between the q-delta model and the quantified stroma area (*p* = 0.003 and *p* = 0.04) and density (*p* = 0.04 and *p* = 0.03) in D1 and D3 (external validation), respectively, whereby low q-delta tumors exhibited larger stromal areas and lower cellular density compared to high q-delta tumors (Figure 2).

### 2.6. Association between q-delta and Clinical Outcome

**Figure 3:**
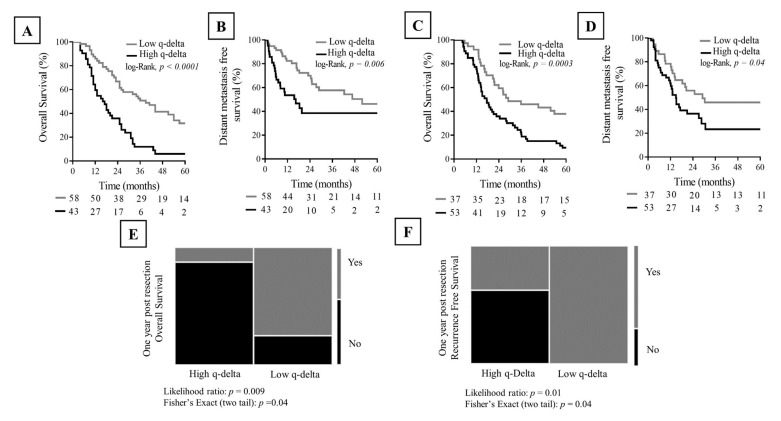
Kaplan Meier analysis shows model-identified q-delta association with the clinical outcome in the training set D1 (**A**,**B**) and the internal validation set (**C**,**D**). Contingency plot shows the association between the q-delta and clinical outcome in the external validation set (**E**,**F**).

In D1, q-delta showed a significant association with the clinical outcome, whereby patients with low q-delta tumors experienced improved overall survival (36 vs. 16.5 months, *p* < 0.0001) and distant metastasis free survival (51.9 vs. 16.4 months, *p* = 0.006) compared to those with high q-delta tumors (Figure 3A,B). The Cox-proportional hazard model identified q-delta as an independent predictor of survival (*p* = 0.004, HR = 2) adjusted for the traditional covariates of survival in localized PDAC: resection margin, lymph node involvement, and receipt of chemotherapeutic regimens (Table 3).

Similarly, in D2, q-delta showed a significant association with the clinical outcome, whereby patients with low q-delta tumors experienced improved overall survival (29 vs. 17.5 months, *p* = 0.0003) and distant metastasis free survival (29.1 vs. 14.9 months, *p* = 0.04) (Figure 3C,D). The Cox-proportional hazard model identified q-delta as an independent predictor of survival (*p* = 0.001, HR = 2.9) adjusted for surgical resection status, resection margin, and lymph node involvement (Table 4).

In D3 (external validation), detailed survival data were not consistently available for all the patients to conduct Kaplan Meier survival analysis. Alternatively, we conducted likelihood ratio and Fisher’s Exact tests, and the results showed that low q-delta patients had a higher likelihood of achieving post resection one-year overall survival (*p* = 0.009, RR = 2.5) and one-year recurrence free survival (*p* = 0.01, RR = 3) when compared to patients with high q-delta tumors. (Figure 3E,F).

## 3. Discussion

In this study, we investigated the potential utility of a commercially available tool to measure a quantitative normalized enhancement feature that could be used to build a predictive model for classification of subtypes of PDAC tumors on pre-therapy pancreatic-protocol CT scans. In the studied cohorts, the q-delta model demonstrated significant accuracy in predicting the qualitative delta class. Furthermore, the q-delta model exhibited differences at the histopathological and the clinical levels, whereby high q-delta tumors were associated with a lower stromal content, higher cellularity, and worse clinical outcome compared to low q-delta tumors. This quantitative classification may help with clinical integration of imaging-based subtypes of PDAC and provides additional insights into the clinical and biological aspects of these subtypes, which can help in developing personalized therapeutic strategies.

Multiple studies have investigated the association between PDAC enhancement and clinical outcomes [10,11,16,17,18]. Previously, we identified subtypes of PDAC tumors from diagnostic CT scans and demonstrated that in localized and metastatic human PDAC, visually scoring the change in enhancement on CT scans at the interface between the tumor and parenchyma (delta) was biologically and clinically relevant. However, in some cases, a visual classification can be challenging and subjective, and a quantitative tool can yield more objective and standardized classification. The proposed quantitative analysis pipeline has two advantages: First, a 3D-contouring approach reduces the selection bias associated with a single region of interest placement and addresses the tumor heterogeneity. Second, using a voxel-based normalized-enhancement evaluation (qEASL) method allows for a detailed evaluation of the intrinsic enhancement features of the tumor relative to a normal reference region compared to using only the mean Hounsfield units.

The enhancement patterns of PDAC tumors are related to their dense desmoplastic stromal reaction and to the arterial supply of the pancreas [5,8,18,19]. We previously reported that mass transport properties, derived from CT scans, correlated with the delivery of and response to gemcitabine and radiation. These mass transport properties were directly measured using the change in the mean Hounsfield units from the pancreatic protocol CT scans from the AR to PV phases, and these changes were referenced to the changes in the NPP for the same phases. These measurements of mass transport provided a normalized area under the enhancement curve (nAUC) that was associated with overall survival. Here, we observed that using the NPP as a normalization reference to the voxel-wise enhancement measurement of qEASL at each phase of the pancreatic protocol CT yielded better sensitivity and specificity in identifying the imaging-based subtypes [20,21]. This q-delta method provides an analogous method to deriving nAUC with some advantages, as it uses a voxel-based approach to map the volumetric tumor enhancement within 1 standard deviation of the normalizing reference, which properly addresses the intratumor heterogeneity. An advantage of the q-delta approach is that it is commercially available and integrated into clinical workstations. Further emphasizing the clinical and biological relevance of the q-delta approach, we observed a significant association between the stromal content and the q-delta as we did for the qualitative delta previously, whereby low q-delta tumors were more likely to have larger stromal areas and lower cellular density. This finding again suggests a role of the stroma in tumor progression, response, and metastasis [22].

This study has some limitations. First is the retrospective nature of the study, as it spanned more than 12 years, during which CT scanners and contrast injection techniques have evolved. We remedied that by including patients who underwent pancreatic-protocol CT scans, which remained essentially unchanged over the time period in terms of the timing of the image acquisitions [23,24,25,26]. Second, the segmentation process may also pose a limitation. To address that, we utilized a validated semi-automatic 3D segmentation tool that required minimal user interaction [12]. This tool was demonstrated to be valuable in delineating iso-attenuating tumors, while guided by the established secondary signs such as mass effect, interrupted duct, and atrophic distal parenchyma [27]. Additionally, the reproducibility of the reported findings were confirmed by the significant inter- and intrarater agreement rates we obtained. Third, patients in the external validation set (D3) had a predominantly more aggressive PDAC tumor phenotype in terms of the T stage, nodal involvement, and margin status, compared to the patients we used to train (D1) and internally validate (D2) the model. This disproportion might have influenced the model ability to elicit more significant differences in the clinical outcome in D3. The future directions for this work include identifying whether the molecular subtypes of PDAC associate with these imaging-based phenotypes, characterizing the stromal and immune cellular populations, evaluating the dynamic changes in these phenotypes in response to therapy, exploring the potential use of this voxel-based quantitative tool to characterize pre-malignant pancreatic lesions, such as intraductal papillary mucinous neoplasms (IPMNs), to differentiate benign from malignant mucinous pancreatic cysts, and finally, applying a similar classification to other hepatobiliary cancers, notably intrahepatic cholangiocarcinoma [28,29,30,31].

## 4. Materials and Methods

### 4.1. Patients

**Figure 4:**
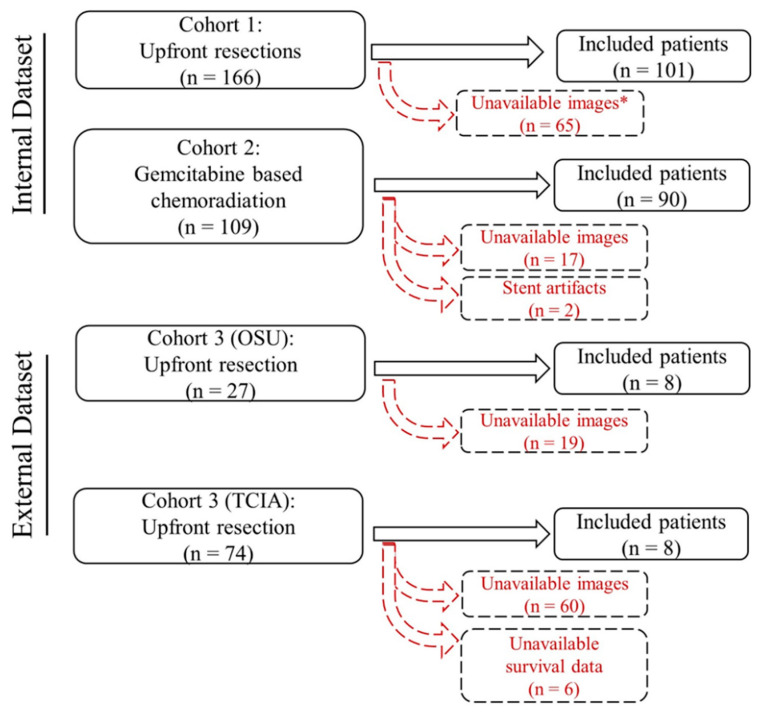
Flow diagram of the included patients in the three studied cohorts. * Unavailable images: pancreas protocol CT scans are either unavailable or missing one or more phases. Abbreviations: OSU: Ohio State University; TCIA: The Cancer Imaging Archive.

After obtaining Institutional Review Board approval (PA14-0646), we retrospectively evaluated patients with resectable or borderline resectable PDAC [32], who had undergone pre-therapy pancreatic protocol CT scans (between 2000 and 2012) at the MD Anderson Cancer Center (MDACC). We identified two separate cohorts: (1) A cohort of 101 patients who underwent curative intent surgical resection without receiving any prior therapeutic regimens (D1), (2) A cohort of 90 patients who received neoadjuvant gemcitabine-based chemoradiation as part of two phase II trials (D2). As an external validation, we evaluated a third cohort (D3) comprised of 16 patients who underwent upfront surgical resection as follows: eight patients from Ohio State University (OSU) (between 2008 and 2014) and eight patients from the publically available Cancer Imaging Archive database [33,34]. A consort diagram of the studied patients is shown in Figure 4.

### 4.2. CT Acquisition

CT scans were acquired using the pancreatic protocol, which is a diagnostic test for patients with PDAC where iodine-based contrast is injected intravenously [19]. A fixed-time delay technique was used for scans obtained before 2006, which consisted of a pre-contrast, an arterial (AR) phase (40 s after starting contrast infusion), and a portovenous (PV) phase (65–70 s after starting contrast infusion). Scans obtained since 2006 used the bolus tracking technique, whereby a value of 100 HU in the aorta triggers the countdown to start the AR phase scan, followed by the PV phase. Scans were acquired using a multi-slice CT system (tube voltage: 120–140 kVp and slice thickness range: 1.25–5 mm (mean = 2.5 mm, SD = 0.47 mm).

### 4.3. CT Analysis: Normalized Area under the Curve (nAUC)

To calculate nAUC, we followed the previously described steps [5]. Briefly, we systematically sampled and measured the HU of both PDAC tumor (HUm) and normal pancreas (HUp) over non-contrast (NC), AR, and PV phases and plugged the measurement in the following equation to obtain the quantitative nAUC readouts:

$$nAUC=\frac{5\left(\mathrm{HUmAR}-\mathrm{HUmNC}\right)+35\left(\left(\mathrm{HUmAR}-\mathrm{HUmNC}\right)+\left(\mathrm{HUmPV}-\mathrm{HUmNC}\right)\right)}{5\left(\mathrm{HUpAR}-\mathrm{HUpNC}\right)+35\left(\left(\mathrm{HUpAR}-\mathrm{HUpNC}\right)+\left(\mathrm{HUpPV}-\mathrm{HUpNC}\right)\right)}$$

### 4.4. CT Analysis: Qualitative Delta Scoring

Qualitatively, two independent radiologists blinded of the outcome (ET = 28 years of experience, PB = 19 years of experience) scored the PDAC tumors on baseline (pre-therapy) CT scans based on conspicuity, shape, and extension into low and high delta groups.

### 4.5. CT Analysis: Quantitative Normalized Enhancement (q-delta)

**Figure 5:**
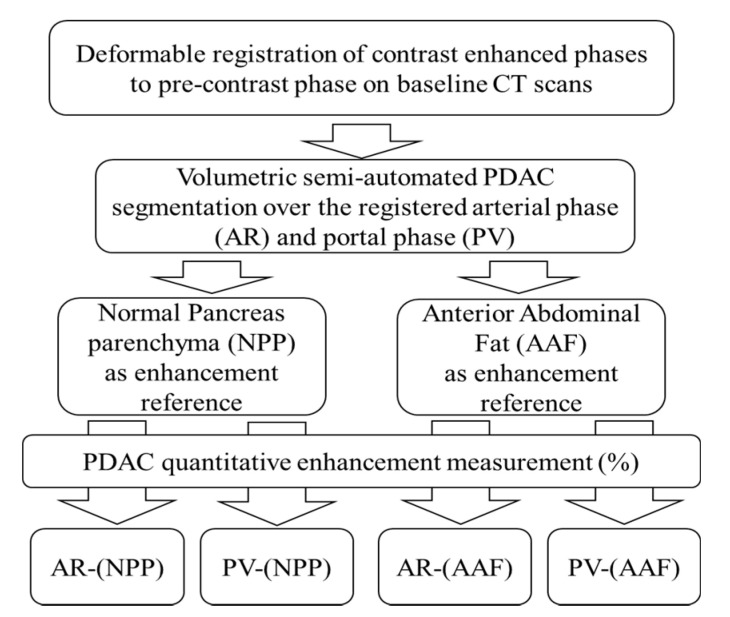
Image analysis pipeline yielded four different quantitative enhancement measurements.

**Figure 6:**
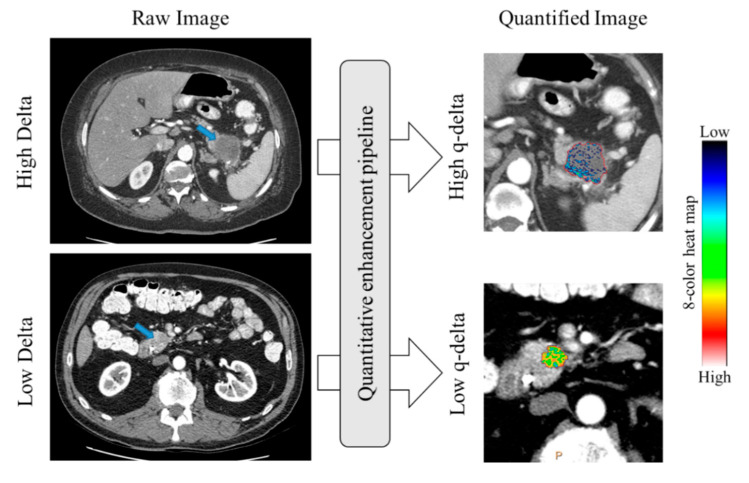
Quantification (q-delta) of pancreatic ductal adenocarcinoma (PDAC) biophysical subtypes. The heterogeneity within PDAC can be visualized using quantitative color mapping.

We used the Philips IntelliSpace portal 8 work station (Multi-Modality Tumor Tracking (MMTT) (Philips Healthcare, Best, NL, USA) to perform our quantitative measurements. First, we used deformable registration to register AR phase scans and PV phase scans separately to the corresponding pre-contrast scan to subtract the background enhancement. Second, we used a semi-automatic segmentation tool to volumetrically contour the tumor, while avoiding the pancreatic duct, surrounding fat, enclosing blood vessels, and metal stents artifacts. We selected two separate regions (~1 cm^3^) over the normal-pancreatic-parenchyma (NPP) and anterior abdominal fat (AAF) to serve as normalization references. Then, we obtained the normalized enhancement (NE) values of the tumor in the AR and PV phases separately, while referencing NPP and AAF (Figure 5). The obtained values represent the percentage of tumor voxels with enhancement values measuring 1 SD over the mean enhancement in the reference region [13]. An 8-color map was overlaid to visualize the tumor enhancement heterogeneity (Figure 6). Contours were reviewed by an independent radiologist blinded of the outcome (ET = 28 years of experience).

### 4.6. Inter- and IntraRater Agreement

To evaluate interrater agreement, two trained researchers independently contoured the PDAC tumors in 30 cases from D1 and D2, followed by generating the NE values while using NPP as a normalization reference. To evaluate intrarater agreement, repeat measurements were performed for the same 30 cases (>3-week interval), followed by generating the NE values while using NPP as a normalization reference. We used the Dice Similarity Coefficient (DSC) to assess inter- and intrarater contour reproducibility and the intraclass correlation coefficient (ICC) to test inter- (two-way random effects, absolute agreement) and intrarater (two-way mixed effects, absolute agreement) NE measurements stability and reported the agreement rates accordingly [35,36].

### 4.7. Histopathological Analysis

We stained the histologic sections of treatment-naïve PDAC tissue (D1 = 59, D3 = 8) with hematoxylin and eosin. We digitally scanned the slides (20x) using an Aperio scanner and used Definiens Architect 2.7 and Visiopharm 9.4 to quantify the stroma area and the cellular density, using machine learning-based tissue and nuclear segmentation algorithms.

### 4.8. Statistical Analysis

We used D1 (*n* = 101) as a training set, and D2 (*n* = 90) and D3 (*n* = 16) as separate validation sets. We performed univariate logistic regression analysis to correlate the quantified NE values with the qualitative delta scoring. A backwards elimination process was used to identify the most significant measurements, followed by creating a multivariate logistic regression model (q-delta model). We used receiver operating characteristic (ROC) analysis to assess the predictive capabilities of the model. We used Kaplan Meier analysis, the Cox proportional hazard model, and likelihood ratio for survival analysis, Spearman’s coefficient to evaluate the relationship between the NE values and nAUC, and a t-test for the q-delta association with the stromal content. Statistical analyses were conducted using JMP Pro 15 (SAS Institute, Cary, NC, USA) and Prism 8 (Graphpad). All tests were two-sided, and a *p*-value ≤ 0.05 was considered significant.

## 5. Conclusions

We used machine learning and quantitative approaches on a multi-institutional dataset to build a clinically relevant model that can quantitatively identify subtypes of PDAC from routine CT scans. As previous studies have demonstrated, these imaging-based subtypes have distinct biophysical properties and clinical outcomes [10]. In both training and validation sets, the model demonstrated a significantly high predictive performance. The model-based classification (q-delta) was demonstrated to be associated with the clinical outcome and the stromal heterogeneity of the PDAC tumors. We believe that the proposed model has the potential to be incorporated into clinical practice, and future studies aimed at validation are warranted.

## Figures and Tables

**Table 1:** Patients’ characteristics obtained from the MD Anderson Cancer Center (MDACC) data sets and the external validation data sets from Ohio State University (OSU) and The Cancer Imaging Archive (TCIA).

Characteristic	MDACC Data		External Validation Data	
	D1	D2	D3	
	Training Set (*n* = 101)	Validation Set (*n* = 90)	OSU (*n* = 8)	TCIA (*n* = 8)
	**No. (%)**	**No. (%)**	**No. (%)**	**No. (%)**
Age (median, range)	64 (25–85)	63 (42–82)	67.5 (50–88)	65 (34–80)
Sex				
Women	46 (47)	38 (42)	3 (27)	3 (37)
Men	55 (53)	52 (58)	5 (63)	5 (63)
Pathological T stage				
T1	21 (21)	24 (37)	0 (0)	0 (0)
T2	67 (66)	35 (54)	0 (0)	0 (0)
T3	12 (12)	6 (9)	8 (100)	8 (100)
T4	1 (1)	-	0 (0)	0 (0)
Pathological N stage				
Negative (N0)	19 (19)	34 (52)	-	1 (12)
Positive (N1)	30 (30)	22 (34)	3 (38)	7 (88)
Positive (N2)	52 (51)	9 (14)	5 (62)	-
Overall Stage				
IA	8 (8)	15 (23)	-	-
IB	9 (9)	16 (25)	-	-
IIA	2 (2)	3 (5)	-	1 (12)
IIB	30 (30)	22 (34)	3 (38)	7 (88)
III	52 (51)	9 (14)	5 (62)	-
IV	-	-	-	-
Surgery				
Yes	101 (100)	65 (72)	8 (100)	8 (100)
No	-	25 (28)	-	-
Surgical margin				
Negative (R0)	87 (86)	62 (95)	2 (25)	5 (62)
Positive (R1)	14 (14)	3 (5)	6 (75)	3 (38)
Adjuvant chemotherapy				
Yes	76 (75)	-	6 (75)	NA*
No	25 (25)	-	2 (25)	NA
Adjuvant chemoradiation				
Yes	33 (33)	-	-	NA
No	68 (67)	-	8 (100)	NA

NA: Not Available.

**Table 2:** Univariate logistic regression analysis of the measured normalized enhancement values using the training data set, followed by multivariate logistic regression using both AR-(NPP) and PV-(NPP) to build and test the predictive model.

Variable	*p*-Value	AUC (95% CI)	Sensitivity (%)	Specificity (%)	Accuracy (%)	Misclassification (%)
**Univariate logistic regression**						
Enhancement AR-(NPP)	<0.0001	0.94 (0.90–0.99)	89	85	87	13
Enhancement PV-(NPP)	<0.0001	0.91 (0.84–0.97)	89	81	85	15
Enhancement AR-(AFF)	<0.0001	0.73 (0.63–0.84)	65	74	70	30
Enhancement PV-(AFF)	0.0003	0.70 (0.60–0.81)	54	78	68	32
**Multivariate logistic regression**						
Training set (q-delta)	<0.0001	0.95 (0.91–0.99)	91	89	91	9
Internal validation set (q-delta)	<0.0001	0.96 (0.92–1)	100	83	93	7
External validation set (q-delta)	0.03	0.90 (0.69–1)	85	1	93	7

AR: Arterial phase; PV: Portovenous phase; NPP: Normal pancreatic parenchyma; AFF: Anterior abdominal fat.

**Table 3:** Univariate and multivariate analyses of patients who underwent upfront surgery for resectable PDAC (D1).

Characteristic	No. of Patients	Univariate Analysis		Multivariate Analysis	
		HR (95% CI)	*p* Value	HR (95% CI)	*p* Value
q-delta					
High	43	2.6 (1.6–4.1)	<0.0001	2 (1.2–3.4)	0.004
Low	58	-	-	-	-
Age	101	1 (0.9–1.03)	0.3	-	-
Sex					
Male	55	0.69 (0.4–1.07)	0.1	0.8 (0.5–1.3)	0.5
Female	46				
Surgical margin					
Positive (R1)	14	1.6 (0.7–2.8)	0.18	1.3 (0.6–2.5)	0.3
Negative (R0)	87	-	-	-	-
Pathologic N Stage					
N0 vs. N1	49	0.47 (0.21–0.97)	0.04	0.4 (0.2–1.03)	0.06
N1 vs. N2	82	0.6 (0.41–1.1)	0.1	0.7 (0.4–1.1)	0.2
N0 vs. N2	71	0.3 (0.1–0.6)	0.0005	0.3 (0.1–0.6)	0.002
Adjuvant chemotherapy					
Yes	76	0.5 (0.3–0.9)	0.02	0.5 (0.3–0.9)	0.02
No	25	-	-	-	-

**Table 4:** Univariate and multivariate analyses of patients who received gemcitabine-based chemoradiation (D2).

		Univariate Analysis		Multivariate Analysis	
Characteristic	No. of Patients	HR (95% CI)	*p* Value	HR (95% CI)	*p* Value
**q-delta ^(†)^**					
High	52	2.4 (1.4–3.9)	*0.0003*	1.9 (1.1–3.2)	*0.01*
Low	37	-	-	-	-
**Surgery**					
Yes	65	0.13 (0.07–0.2)	*<0.0001*	0.1 (0.06–0.2)	<0.0001
No	25	-	-	-	-
**q-delta ^(††)^**					
High	35	2.5 (1.4–4.6)	*0.0003*	2.9 (1.6–5.5)	*0.001*
Low	30	-	-	-	-
**Age**	65	1.02 (0.9–1.06)	0.16	1 (0.4–7)	0.3
**Sex**					
Male	36	1 (0.62–1.8)	0.7	-	-
Female	29				
**Surgical margin**					
Positive (R1)	62	1.3 (0.4–4.2)	0.6	-	-
Negative (R0)	3	-	-	-	-
**N Stage**					
N0 vs. N1	56	1 (0.5–1.9)	0.8	-	-
N1 vs. N2	31	0.6 (0.3–1.5)	0.3	-	-
N0 vs. N2	41	0.7 (0.3–1.5)	0.3	-	-

^(†)^ Patients with potentially resectable pancreatic cancer; ^(††)^ Patients who underwent surgical resection.

## Supplementary Materials

The following are available online at https://www.mdpi.com/2072-6694/12/12/3656/s1, Figure S1. The probability equation used to classify the response level into high versus low q-delta. Figure S2. The association between nAUC and tumor enhancement in D1: training set (A,B), D2: internal validation set (C,D) and D3: external validation set (E,F). The association between nAUC and q-delta in the three studied cohorts (G). Table S1. Intra- and inter-rater agreement of DSC and ICC in 30 patients.
