# Supplementary material for: Predictive Modeling for Voxel-Based Quantification of Imaging-Based Subtypes of Pancreatic Ductal Adenocarcinoma (PDAC): A Multi-Institutional Study
Source: Cancers (Basel). 2020 Dec 5;12(12):3656. doi: 10.3390/cancers12123656 (PMC7762105; doi:10.3390/cancers12123656)
Supplement: Supplementary file 1 [file cancers-12-03656-s001.pdf]

Suplementary Materials: Predictive Modeling for Voxel-based Quantification of Imaging-based Subtypes of Pancreatic Ductal Adenocarcinoma (PDAC): A Multi-Institutional study

Mohamed Zaid, Lauren Widmann, Annie Dai, Kevin Sun, Jie Zhang, Jun Zhao, Mark Hurd, Gauri R Varadhachary, Robert A. Wolff, Anirban Maitr, Matthew HG Katz, Joseph M Herman[1], Huamin Wang, Michael V Knopp, Terence M Williams, Priya Bhosale, Eric P Tamm and Eugene J Koay

$$linear\ combination\ of\ response\ level\ (lin) = 2.45 + [\ -0.85 \times PV\text{"NPP "}] + [\ -0.41 \times AR\ NPP]$$
$$propability\ of\ high\ q\text{-}delta = 1/((1 + Exp\ (-lin)))$$
$$propability\ of\ lwo\ q\text{-}delta = 1/((1 + Exp\ (lin)))$$

Figure 1. The probability equation used to classify the response level into high versus low q-delta. Abbreviations: AR NPP: enhancement at arterial phase normalized to normal pancreas parenchyma; PV NPP: enhancement at portovenous phase normalized to normal pancreas parenchyma.

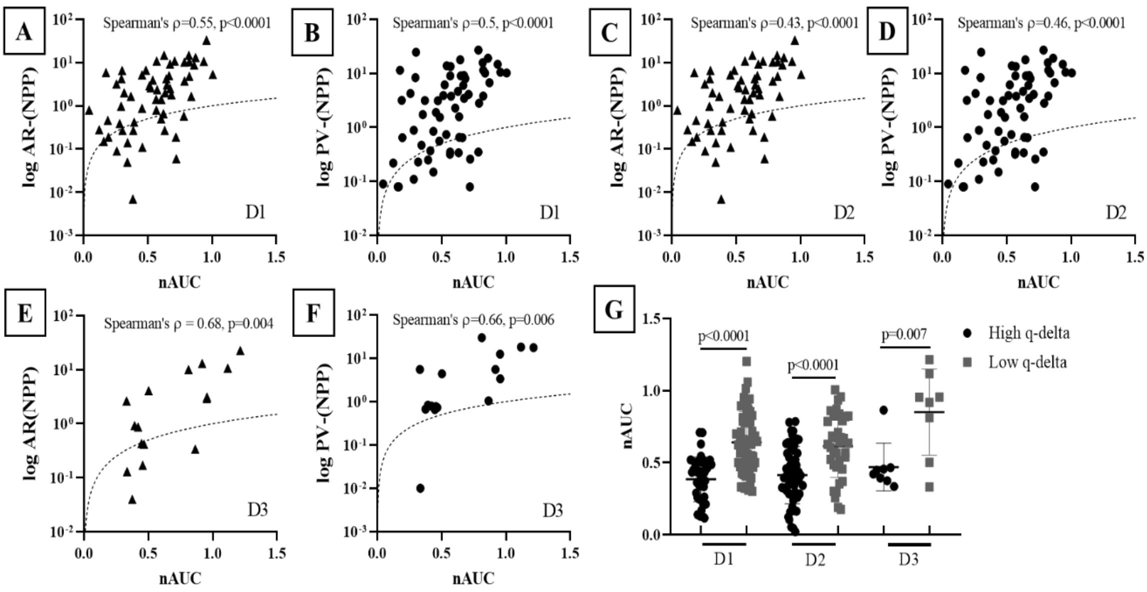

Figure 2. The association between nAUC and tumor enhancement in D1: training set (**A**,**B**), D2: internal validation set (**C**,**D**) and D3: external validation set (E-F). The association between nAUC and q-delta in the three studied cohorts (G). Abbreviations: (AR)-NPP: enhancement at arterial phase normalized to normal pancreas parenchyma nAUC: normalized area under the curve; (PV)-NPP: enhancement at portovenous phase normalized to normal pancreas parenchyma.

Table S1. Intra- and inter-rater agreement of DSC and ICC in 30 patients.

Variable			Average	Lower 95%CI	Upper 95%CI	Agreement Interpretation
Dice similarity coefficient (DSC)	Intra-observer	All	0.9	0.88	0.92	Good
		High q-delta	0.93	0.9	0.96	Good
		Low q-delta	0.87	0.85	0.89	Good
	Inter-observer	All	0.86	0.82	0.89	Good
		High q-delta	0.89	0.82	0.95	Good
		Low q-delta	0.83	0.80	0.86	Good
Intraclass Correlation Coefficient (ICC)	Intrarater (AR-NPP)		0.95	0.92	0.97	Excellent
	Interrater (AR-NPP)		0.84	0.71	0.91	Good
	Intrarater (PV-NPP)		0.93	0.87	0.96	Excellent
	Interrater (PV-NPP)		0.90	0.82	0.95	Excellent

AR: Arterial phase ; PV: Portovenous phase; NPP: Normal pancreatic parenchyma.

© 2020 by the authors. Licensee MDPI, Basel, Switzerland. This article is an open access article distributed under the terms and conditions of the Creative Commons Attribution (CC BY) license (http://creativecommons.org/licenses/by/4.0/).
